# A Ball Bar Measurement Scheme with Single-Axis Rotation Modes for Position-Independent Geometric Error Identification in Dual Rotary Table Five-Axis Machine Tools

**DOI:** 10.3390/s26123789

**Published:** 2026-06-14

**Authors:** Jinlong Zhang, Hongtao Yang, Zhaoyao Shi

**Affiliations:** 1 Beijing Engineering Research Center of Precision Measurement Technology and Instruments, Beijing University of Technology, Beijing 100124, China; zhangjinlong063@126.com; 2 School of Mechatronics Engineering, Anhui University of Science and Technology, Huainan 232001, China; lloid@163.com

**Keywords:** five-axis machine tool, rotating axes, PIGEs, ball bar, error identification

## Abstract

**Highlights:**

- A ball bar method is proposed to identify eight position-independent geometric errors (PIGEs) of dual rotary table five-axis machine tools, with four measurement models established.
- After error compensation, the maximum individual error is reduced from 144.53 μm to 7.72 μm, with an overall error reduction rate of 79.48%.
- This method addresses key challenges in PIGE identification, improves machine tool precision, and offers a practical solution with broad applicability.

**Abstract:**

Position-independent geometric errors (PIGEs) of rotary axes are critical factors limiting the machining accuracy of five-axis machine tools. Aiming at the accurate identification of rotary-axis PIGEs for dual rotary table five-axis machine tools, this study proposes an improved double ball bar (BB) measurement scheme. This scheme features excellent decoupling performance, convenient operation and high efficiency: complete error decoupling is realized via independent single-axis rotary measurement; a standard fixed-length ball bar is adopted without any auxiliary fixtures to effectively reduce setup-induced errors; a non-iterative analytical algorithm based on circular eccentricity fitting is developed to accurately identify all geometric errors of rotary axes. Based on homogeneous coordinate transformation theory and the fundamental BB measurement principle, mathematical models that correlate rotary-axis PIGEs with BB length deviations are established for four designed measurement modes. By constraining only one single rotary axis to move while fully locking all other axes during each test, the proposed method enables BB measurement data to exclusively reflect the geometric errors of the tested rotary axis, thereby fundamentally eliminating geometric error coupling induced by multiple axes. Subsequently, the correlation between PIGEs and BB length variations is quantitatively analyzed via numerical simulation. On this basis, an analytical PIGE identification strategy is developed using circular eccentricity fitting of measured BB trajectory data. Taking a typical BC-type dual rotary table five-axis machine tool as the experimental platform, all eight rotary-axis PIGEs are successfully identified and compensated. Experimental results demonstrate that the maximum positional error is reduced from 144.53 μm to 7.72 μm, achieving an overall accuracy improvement rate of 79.48%. The proposed method enables high-precision PIGE decoupling and identification, effectively improves the machining precision of five-axis machine tools, and exhibits good applicability for dual rotary table machine tools, providing a reliable alternative for geometric error identification in five-axis machining systems.

## 1. Introduction

Five-axis machine tools realize complex curved surface machining via multi-axis coordinated motion and are widely applied in high-end manufacturing fields such as aero-engine blades and automotive precision components [1,2]. Machining accuracy is affected by geometric errors, cutting errors, thermal errors and servo errors [3,4]. In accordance with multiple ISO standards [5,6,7], the rotary-axis geometric errors of dual rotary table five-axis machine tools are classified into eight PIGEs and twelve position-dependent geometric errors (PDGEs) [8]. Generated during assembly and remaining stable throughout the service life of machine tools, PIGEs cannot be measured directly and must be solved indirectly via error identification models.

Available geometric error measurement techniques include double ball bar (BB) [9,10,11,12,13], laser tracker [14,15,16], laser tracer [17,18], touch-trigger probe [19,20], R-test device [21,22] and cutting trial experiments [23,24]. From an industrial application perspective, laser trackers are limited by high procurement costs and weak anti-interference performance under variable workshop temperature and vibration conditions; laser tracers, by contrast, offer unique capabilities such as sub-micrometer accuracy and real-time target tracking, which enable high-precision geometric error measurement in complex dynamic scenarios. However, they are generally more expensive and require controlled environmental conditions, limiting their widespread use in routine industrial calibration; R-test devices require customized special fixtures and tedious pre-calibration, which significantly extends preparation duration; measurements using touch-trigger probes need complicated multi-axis programming and exhibit low detection efficiency; cutting trials are destructive and consume costly blank workpieces, making rapid on-machine geometric calibration impossible. By contrast, the double ball bar features low cost, straightforward operation and excellent measurement repeatability, making it the most widely used measuring instrument.

Existing BB-based identification approaches are categorized into multi-axis synchronous measurement and single-axis measurement in terms of motion control, and both categories feature certain technical limitations. Tsutsumi et al. [25,26] employed multi-axis synchronous circular measurement, where multi-axis motion introduces coupled errors originating from translational axes. Yang [27] built a Jacobian-matrix-based model that still depends on multi-axis movement and iterative computation. Lee [10] optimized measurement paths but required extra auxiliary blocks mounted on the BB, triggering additional setup errors. Although Xiang [28] locked translational axes, synchronous rotation of two rotary axes was still necessary under partial measurement conditions. Jiang [29] adopted single-axis rotation but had to fit extension rods and reassemble the BB repeatedly, which increases setup-induced errors.

A comprehensive literature review reveals that conventional methods are plagued by linear-axis geometric error coupling when identifying rotary-axis PIGEs, alongside other drawbacks such as dependence on auxiliary fixtures, mandatory adjustment of ball-bar length and unstable iterative algorithms. At present, no available BB measurement approach can simultaneously achieve full locking of translational axes, fixture-free measurement, fixed-length BB deployment and non-iterative complete identification of all eight PIGEs in a single measurement campaign.

To address the above-mentioned research limitations, this study proposes an innovative BB-based identification method targeting BC-type dual rotary table five-axis machine tools, which possesses three prominent advantages over existing technologies: (1) Four dedicated single-axis measurement trajectories are designed with all linear axes fully clamped to fundamentally eliminate linear-axis error coupling during testing. (2) Standard fixed-length BB is adopted across all measurements without auxiliary blocks or extension rods to minimize installation-caused errors. (3) Analytical formulas derived from circle eccentricity fitting of measured data enable simultaneous non-iterative calculation of all eight PIGEs.

The remainder of this paper is structured as follows. Section 2 defines the eight rotary-axis PIGEs. Section 3 establishes kinematic models and derives the mathematical formulas corresponding to the four measurement modes. Section 4 describes practical identification and compensation experiments, and Section 5 summarizes the present work.

## 2. PIGEs Definition for Rotating Axes

**Figure 1:**
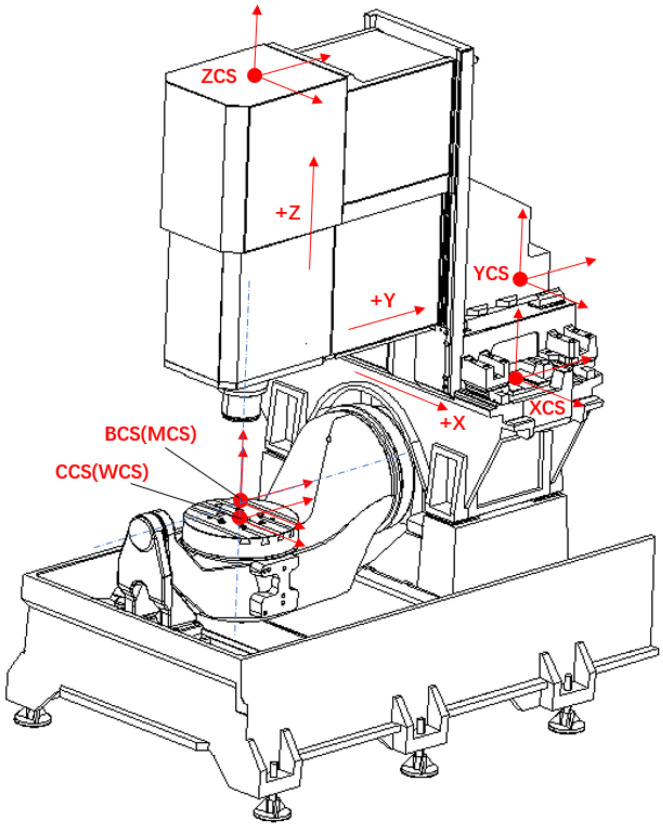
Schematic of five-axis machine tool.

In this paper, error analysis is conducted with the BC dual rotary table five-axis machine tool as the research object. Axes B and C are located on the workpiece side: the B-axis rotary table is fixed to the bed by a support base, and the C-axis rotary table is stacked on it. The X-, Y-, and Z-axis guide rails are on the tool side. The structure and coordinate systems of the machine tool are illustrated in Figure 1.

**Figure 2:**
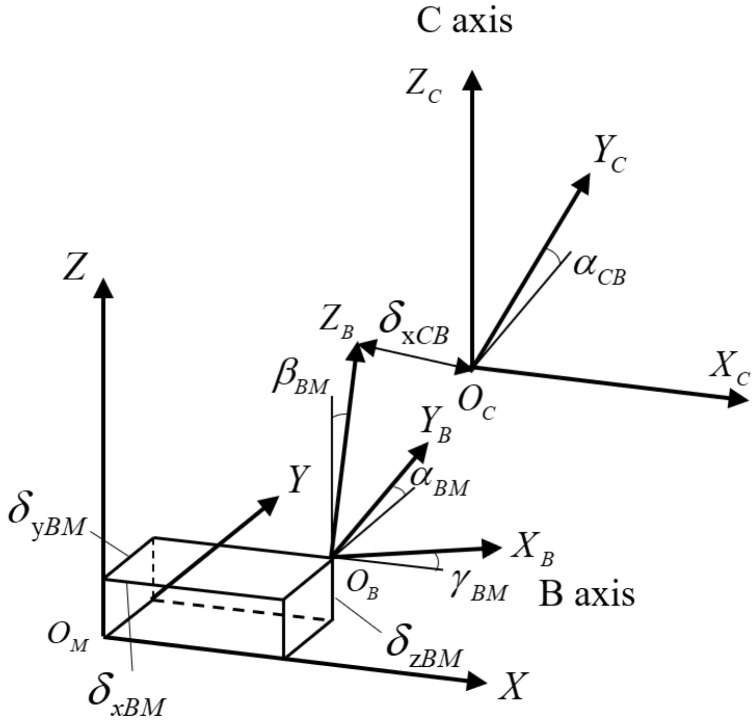
The 8 PIGEs in relative form.

The position-independent geometric errors (PIGEs) of rotary axes in five-axis machine tools are mainly described via two approaches, namely the absolute representation and the relative representation. Both approaches possess solid theoretical foundations and excellent engineering applicability, and have been widely adopted in geometric error modeling and calibration for five-axis machine tools with various configurations. Specifically, the absolute representation describes rotary-axis geometric errors with reference to the machine’s global fixed coordinate system. It is applicable to general error modeling for various machine tool types. In contrast, the relative representation builds error models using the local coordinate systems of adjacent moving axes. This method conforms to the hierarchical assembly features and error propagation laws of machine tools [7,30]. For the BC-type dual rotary table five-axis machine tool studied in this work, the C-axis is mounted on the B-axis. During operation, the spatial pose of the C-axis changes synchronously with the rotation of the B-axis, creating inherent hierarchical assembly constraints between the two axes. Given this structural feature, this paper adopts the relative representation based on the local coordinate systems of adjacent axes to define and model the eight PIGEs of the B and C axes. This method can accurately reflect the actual generation and propagation mechanisms of shaft system errors and clearly describe the assembly geometric deviations in the BC dual axes. The schematic diagram of the corresponding errors is presented in Figure 2.

The details are as follows: The position errors of the B-axis with respect to the machine bed are denoted as $\delta_{xBM}$, $\delta_{yBM}$ and $\delta_{zBM}$ along the X, Y and Z axes, respectively. Rotating with respect to the bed, the B-axis exhibits rotation errors $\alpha_{BM}$, $\beta_{BM}$ and $\gamma_{BM}$ about the X, Y and Z axes, respectively. Along the X-direction, the C-axis rotation and position error relative to the B-axis are designated $\alpha_{CB}$ and $\delta_{xCB}$, respectively.

## 3. Identification Model of the PIGEs of the Rotation Axes

### 3.1. Kinematics Model

**Figure 3:**
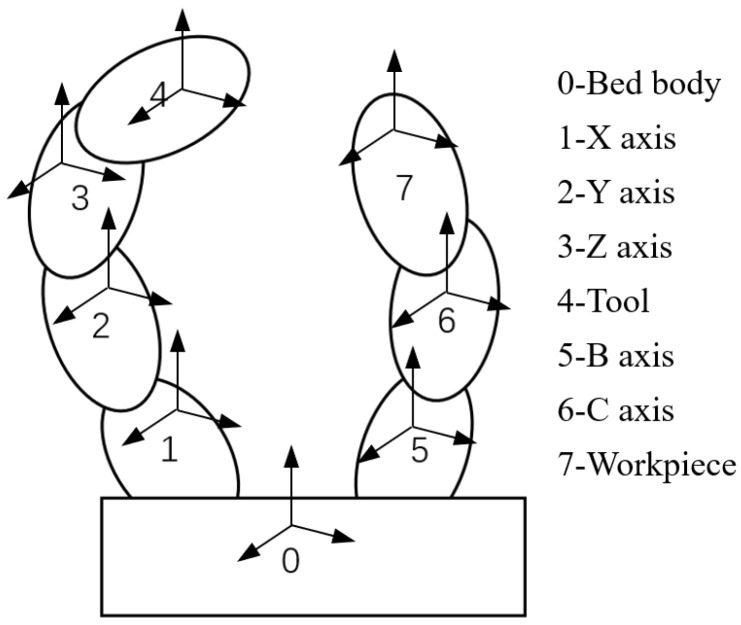
Topological structure diagram of the machine tool.

Figure 3 depicts the topological structure of the machine tool adopted. The machine tool’s kinematic chain is split into two branches: one for the workpiece and one for the tool. Depicted on the left is the tool kinematic sequence (Tool → Z → Y → X → Bed); the workpiece sequence (Workpiece → C → B → Bed) is shown on the right.

Analysis shows that the transformation matrix of ball $O_{W}$ from the workpiece frame to the machine frame is denoted as PMOW:

(1) PMOw=TMB⋅TBC⋅PCOw

(2) TBC=D1δxCBD4αCBD6C

(3) TMB=D1δxBMD2δyBMD3δzBMD5βBMD6γBMD4αBMD5B

The matrix of the tool ball $O_{T}$ in the machine coordinate system (MCS) is denoted as PMOT:

(4) PMOT=TMX⋅TXY⋅TYZ⋅PZOT=x,y,z,1

(5) L2=R+ΔR2=PMOT−PMOw

where THG denotes the transformation matrix converting the G-axis frame to the H-axis frame (similar expressions are used for other coordinate transformations). In the BB setup, the ball connected to the spindle is marked as $O_{T}$, and the one connected to the worktable is marked as $O_{W}$. The total length of the BB, denoted as L, is the distance between $O_{T}$ and $O_{W}$. R is the theoretical length of the BB, and ΔR is its length variation. The nominal translational displacements of the machine tool axes are assigned the parameters x, y and z, while C and B represent the angular parameters for the two rotational axes. The details of each homogeneous coordinate transformation matrix (HCTM) are presented in Table 1. Notation convention: ET stands for the error term, and RA denotes the rotation angle. Specifically, $S_{C}$ represents sin(C), $C_{C}$ represents cos(C) and analogous notations apply to other trigonometric functions.

### 3.2. Measurement Modes for the PIGEs of the Rotating Axes

**Figure 4:**
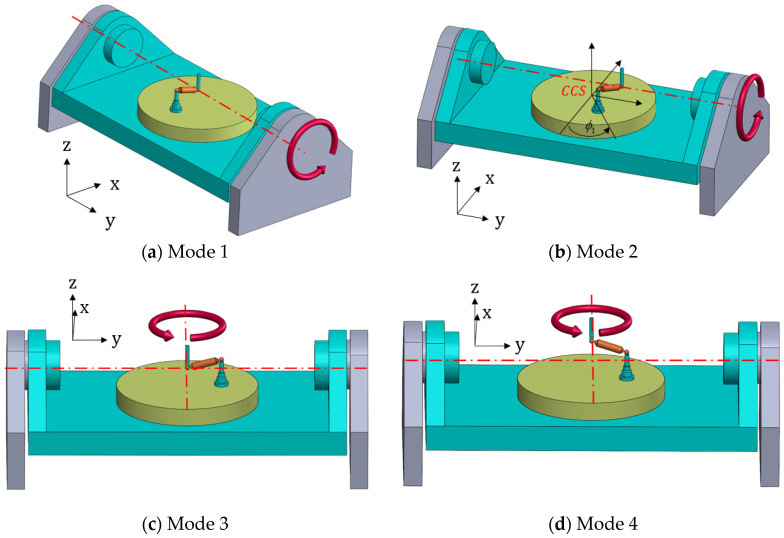
Measurement modes for the PIGEs.

To ensure that the BB measurement data reflects only the PIGEs of the target rotary axis, this paper controls only one of the rotating axes to rotate during each PIGE measurement process, while the other four axes remain stationary. This single-axis strategy physically isolates the measurement from the geometric errors of other participating axes, thereby eliminating the need to model or separate these coupled errors during identification. There are two distinct measurement modes for each rotary axis, which not only facilitates the testing process but also enhances the precision of the measurement results. The four measurement modes are displayed in Figure 4.

The tool ball $O_{T}$ is located at the intersection of the B- and C-axes, with the workpiece ball $O_{W}$ initially positioned on the negative X-axis of the Machine Coordinate System (MCS), as shown in Figure 4a. During measurement, $O_{T}$ remains fixed; only the B-axis rotates, driving $O_{W}$ to revolve about the B-axis.

Figure 4b presents Mode 2. Driven by the C-axis turntable, the workpiece ball $O_{W}$ rotates counterclockwise from the MCS origin to a predefined angle $\phi_{1}$, whereas $O_{T}$ is fixed on the positive Y-axis of MCS. In the measuring process, the B-axis is the sole rotating component, and the rotation makes $O_{W}$ revolve around the B-axis with $O_{T}$ stationary.

Figure 4c shows Mode 3. $O_{T}$ is arranged at the intersection of the B- and C-axes, and $O_{W}$ is initially set on the positive Y-axis of MCS. Throughout measurement, $O_{T}$ remains fixed while exclusive rotation of the C-axis drives $O_{W}$ to rotate about the C-axis.

Figure 4d corresponds to Mode 4. Starting from the initial setup of Mode 3, $O_{T}$ is lifted along the positive Z-axis of MCS. During measurement, $O_{T}$ stays stationary, and the rotating C-axis imparts rotary motion to $O_{W}$ around the C-axis.

### 3.3. Identification Model and Numerical Verification for the PIGEs of Rotating Axes

#### 3.3.1. Coordinate Derivation of the Four Measurement Modes

Based on the kinematics model developed in Section 3.1, an error identification model has been established to correspond with the measurement modes depicted in Figure 4.

Mode 1:

Coordinate derivation outline: The origin of the Machine Coordinate System (MCS) lies at the intersection of the B- and C-axes, so tool ball center $O_{T}$ is initially located at $\left(0,0,0\right)$. The initial coordinates of the workpiece ball $O_{W}$ in the CCS are designated as PCOW=−q0R, 0, h0, 1T, where $q_{0}$ is the adjustment coefficient and $h_{0}$ is the distance along the Z-axis between the workpiece ball and the origin in CCS. Substituting PCOW into Equation (1) PMOw=TMB⋅TBC⋅PCOw, where TMB and TBC are composed of the matrices listed in Table 1. Since the PIGEs are small quantities (on the order of $10^{-3}$ rad or $10^{-5}$ m), only the first-order terms of the error parameters are retained in the matrix chain multiplication, and all second-order and higher product terms are neglected. After expansion and simplification, the coordinates of the workpiece ball shown in Equation (6) are obtained.

(6) PMOw=δxBM+δxCB⋅CB+h0⋅SB+βBM⋅CB−R⋅q0⋅CB−βBM⋅SBδyBM−h0⋅αCB+αBM⋅CB−rBM⋅SB−R⋅q0⋅rBM⋅CB+αBM⋅SBδzBM−h0⋅−CB+βBM⋅SB−δxCB⋅SB+R⋅q0⋅βBM⋅CB+SB1

During measurement, the spatial position of the tool ball remains unchanged, so PMOT is kept at the origin in MCS. By substituting PMOW and PMOT into Equation (5), the length data $L_{1}$ measured by the BB can be obtained:

(7) $$\begin{array}{l} {\left(L_{1}\right)}^{2}={\left[\delta_{xBM}+\delta_{xCB}\cdot C_{B}+h_{0}\cdot \left(S_{B}+\beta_{BM}\cdot C_{B}\right)-R\cdot q_{0}\cdot \left(C_{B}-\beta_{BM}\cdot S_{B}\right)\right]}^{2}+\\ {\left[\delta_{yBM}-h_{0}\cdot \left(\alpha_{CB}+\alpha_{BM}\cdot C_{B}-r_{BM}\cdot S_{B}\right)-R\cdot q_{0}\cdot \left(r_{BM}\cdot C_{B}+\alpha_{BM}\cdot S_{B}\right)\right]}^{2}+\\ {\left[\delta_{zBM}-h_{0}\cdot \left(-C_{B}+\beta_{BM}\cdot S_{B}\right)-\delta_{xCB}\cdot S_{B}+R\cdot q_{0}\cdot \left(\beta_{BM}\cdot C_{B}+S_{B}\right)\right]}^{2} \end{array}$$

Mode 2:

Coordinate derivation outline: The initial coordinates of the workpiece ball $O_{W}$ in the CCS are defined as PCOW=−q1R, L0⋅m, h0, 1T, where $q_{1}$ and m denote the adjustment coefficients. The tool ball is fixed at PMOT=0, L0, 0, 1T in the MCS. Substituting PCOW into Equation (1) PMOw=TMB⋅TBC⋅PCOw, the matrices TMB and TBC are constructed identically to those in Mode 1. Likewise, only the first-order terms of the error parameters are retained, and all second-order and higher product terms are neglected. After expansion and simplification, the coordinates of the workpiece ball shown in Equation (8) are obtained.

(8) PMOw=δxBM−L0⋅m⋅rBM−αCB⋅SB+δxCB⋅CB+h0⋅SB+βBM⋅CB−R⋅q1⋅CB−βBM⋅SBδyBM+L0⋅m−h0⋅αCB+αBM⋅CB−rBM⋅SB−R⋅q1⋅αBM⋅SB+rBM⋅CBδzBM+L0⋅m⋅αBM+αCB⋅CB−h0⋅−CB+βBM⋅SB−δxCB⋅SB+R⋅q1⋅SB+βBM⋅CB1

During measurement, the spatial position of the tool ball remains unchanged, so PMOT is kept at $\left(0,L_{0},0\right)$. By substituting PMOW and PMOT into Equation (5), the measured length $L_{2}$ from the BB is expressed as Equation (9):

(9) $$\begin{array}{l} {\left(L_{2}\right)}^{2}={\left[\begin{array}{c} \delta_{xBM}-L_{0}\cdot m\cdot \left(r_{BM}-\alpha_{CB}\cdot S_{B}\right)+\delta_{xCB}\cdot C_{B}+\\ h_{0}\cdot \left(S_{B}+\beta_{BM}\cdot C_{B}\right)-R\cdot q_{1}\cdot \left(C_{B}-\beta_{BM}\cdot S_{B}\right) \end{array}\right]}^{2}+\\ \;\;\;\;\;\;\;\;\;\;\;\;{\left[\begin{array}{c} \delta_{yBM}+L_{0}\cdot m-h_{0}\cdot \left(\alpha_{CB}+\alpha_{BM}\cdot C_{B}-r_{BM}\cdot S_{B}\right)-\\ R\cdot q_{1}\cdot \left(\alpha_{BM}\cdot S_{B}+r_{BM}\cdot C_{B}\right)-L_{0} \end{array}\right]}^{2}+\\ \;\;\;\;\;\;\;\;\;\;\;\;{\left[\begin{array}{c} \delta_{zBM}+L_{0}\cdot m\cdot \left(\alpha_{BM}+\alpha_{CB}\cdot C_{B}\right)-h_{0}\cdot \left(\beta_{BM}\cdot S_{B}-C_{B}\right)-\\ \delta_{xCB}\cdot S_{B}+R\cdot q_{1}\cdot \left(S_{B}+\beta_{BM}\cdot C_{B}\right) \end{array}\right]}^{2} \end{array}$$

Mode 3:

Coordinate derivation outline: The initial coordinates of workpiece ball $O_{W}$ in the CCS are defined as PCOW=0, −q2R, h0, 1T, where $q_{2}$ denotes the adjustment coefficient. The tool ball is fixed at the origin of the MCS, i.e., PMOT=0, 0, 0, 1T. For this measurement mode, only the C-axis rotates while the B-axis remains stationary (B=0). Accordingly, the rotation angle B in TMB is set to zero, and the rotation angle C in TBC serves as the variable. Only first-order error terms are retained with all higher-order terms neglected; after algebraic expansion and simplification, the coordinate expression of the workpiece ball is derived as Equation (10):

(10) PMOw=δxBM+δxCB+h0⋅βBM−R⋅q2⋅(SC+CC⋅rBM)δyBM−h0⋅(αBM+αCB)−R⋅q2⋅(rBM⋅SC−CC)δzBM+h0+R⋅q2⋅(βBM⋅SC+CC⋅(αBM+αCB))1

During measurement, the spatial position of the tool ball remains unchanged, so PMOT is fixed at the MCS origin $\left(0,0,0\right)$. Substituting PMOW and PMOT into Equation (5), the measured length $L_{3}$ from the BB is expressed as Equation (11):

(11) $$\begin{array}{l} {\left(L_{3}\right)}^{2}={\left[\delta_{xBM}+\delta_{xCB}+h_{0}\cdot \beta_{BM}-R\cdot q_{2}\cdot \left(S_{C}+C_{C}\cdot r_{BM}\right)\right]}^{2}+\\ \;\;\;\;\;\;\;\;\;\;\;\;{\left[\delta_{yBM}-h_{0}\cdot \left(\alpha_{BM}+\alpha_{CB}\right)-R\cdot q_{2}\cdot \left(r_{BM}\cdot S_{C}-C_{C}\right)\right]}^{2}+\\ \;\;\;\;\;\;\;\;\;\;\;\;{\left[\delta_{zBM}+h_{0}+R\cdot q_{2}\cdot \left(\beta_{BM}\cdot S_{C}+C_{C}\cdot \left(\alpha_{BM}+\alpha_{CB}\right)\right)\right]}^{2} \end{array}$$

Mode 4:

Coordinate derivation outline: The initial coordinates of workpiece ball $O_{W}$ in the CCS remain the same as those defined in Mode 3, i.e., PCOW=0, −q2R, h0, 1T. Since $O_{W}$ is arranged in CCS identically to Mode 3, the coordinate PMOW are given in Equation (10). The tool ball is positioned at PMOT=0, 0, h1, 1T in the MCS, offset by $h_{1}$ along the positive Z-axis of MCS. Only the C-axis rotates while the B-axis remains stationary. By substituting PMOW and PMOT into Equation (5), the measured length $L_{4}$ from the BB is expressed as Equation (12):

(12) $$\begin{array}{l} {\left(L_{4}\right)}^{2}={\left[\delta_{xBM}+\delta_{xCB}+h_{0}\cdot \beta_{BM}-R\cdot q_{2}\cdot \left(S_{C}+C_{C}\cdot r_{BM}\right)\right]}^{2}+\\ \;\;\;\;\;\;\;\;\;\;\;\;{\left[\delta_{yBM}-h_{0}\cdot \left(\alpha_{BM}+\alpha_{CB}\right)-R\cdot q_{2}\cdot \left(r_{BM}\cdot S_{C}-C_{C}\right)\right]}^{2}+\\ \;\;\;\;\;\;\;\;\;\;\;\;{\left[\delta_{zBM}+h_{0}-h_{1}+R\cdot q_{2}\cdot \left(\beta_{BM}\cdot S_{C}+C_{C}\cdot \left(\alpha_{BM}+\alpha_{CB}\right)\right)\right]}^{2} \end{array}$$

In Equations (6)–(12), $S_{B}$ represents sin(B), $S_{C}$ represents sin(C), $C_{B}$ represents cos(B), $C_{C}$ represents cos(C).

#### 3.3.2. Linearization of the BB Length Variation Model

All coordinate transformation and simplification in subsequent identification follow four rules: (1) The homogeneous coordinate transformation theory is applied to map the coordinates through the machine tool kinematic chain (Workpiece → C→ B→ Bed). (2) All geometric errors are small quantities; thus, the approximations sin(ε)≈ε, cos(ε)≈1 are adopted, and second-order and higher product terms (e.g., $\delta_{xBM}\cdot \alpha_{CB}$, $\beta_{BM}^{2}$, etc.) are strictly neglected. (3) The BB length variation satisfies L=R+ΔR with ΔR≪R, permitting the binomial linearization: ${\left(R+\Delta R\right)}^{2}\approx R^{2}+2R\cdot \Delta R$. (4) Only first-order error terms coupled with trigonometric functions of the rotation angles are retained in the final expressions.

Directly substituting raw ball-bar measured data into Equations (7), (9), (11) and (12) results in heavy computational burden. To simplify the BB length models, the following linearization is applied. Let L=R+ΔR denote the measured BB length, where R is the theoretical length and ΔR is the length variation. Since ΔR≪R, the squared length can be linearized using the binomial approximation:

(13) $${\left(R+\Delta R\right)}^{2}=R^{2}+2R\Delta R+{\left(\Delta R\right)}^{2}\approx R^{2}+2R\Delta R$$

The actual coordinates of the workpiece ball PMOW can be decomposed into the ideal coordinates PMOW0 (with zero geometric errors) and the error-induced deviation PMOWe:

(14) PMOW=MPOW0+MPOWe

The tool ball remains stationary during measurement, so its coordinates PMOT are constant. Substituting Equation (14) into the distance formula yields

(15) L2=PMOW−MPOT2=PMOW0−MPOT+MPOWe2    =R2+2PMOW0−MPOTT⋅MPOWe+PMOWe2

where R2=PMOW0−MPOT2. Since PMOWe is a first-order small quantity proportional to the PIGEs, the term PMOWe2 is a second-order small quantity and is neglected. Equating Equations (13) and (15) and eliminating $R^{2}$ yields

(16) ΔR=1RPMOW0−MPOTT⋅MPOWe

Take Mode 1 as a representative example to demonstrate the complete simplification from Equation (7) to $\Delta R_{1}$:

In Mode 1, PMOT=0, 0, 0, 1T. Split PMOW=MPOW0+MPOWe, where PMOW0 denotes ideal coordinates without any PIGEs and PMOWe stands for error-induced small offset extracted from Equation (6). Substitute into distance formula:

(17) L2=PMOW−MPOT2=PMOW0−MPOT+MPOWe2    =PMOW0−MPOT2+2PMOW0−MPOTT⋅MPOWe+PMOWe2

Since R=PMOW0−MPOT and PMOWe2 is high-order infinitesimal and omitted, combine $L^{2}\approx R^{2}+2R\Delta R$ in Equation (13): ΔR=1RPMOW0−MPOTT⋅MPOWe.

Substitute the specific coordinate expression from Equation (6), collect terms of cosB, sinB, eliminate constant terms, and finally obtain the simplified $\Delta R_{1}$ listed in Equation (18).

Derivations for Mode 2, Mode 3 and Mode 4 follow identical decomposition and higher-order small-term truncation rules, so their simplified $\Delta R_{2}$, $\Delta R_{3}$, $\Delta R_{4}$ are directly summarized in Equation (18).

(18) $$\left\{\begin{array}{l} \Delta R_{1}=\cos(B)\cdot (-q_{0}\cdot \delta_{xBM})+\sin(B)\cdot (q_{0}\cdot \delta_{zBM})-q_{0}\cdot \delta_{xCB}\\ \Delta R_{2}=\cos(B)\cdot (-q_{1}\cdot \delta_{xBM}+q_{1}\cdot L_{0}\cdot \gamma_{BM})+\sin(B)\cdot (q_{1}\cdot \delta_{zBM}+q_{1}\cdot L_{0}\cdot \alpha_{BM})-q_{1}\cdot \delta_{xCB}\\ \Delta R_{3}=\cos(C)\cdot (q_{2}\cdot \delta_{yBM})+\sin(C)\cdot (-q_{2}\cdot \delta_{xBM}-q_{2}\cdot \delta_{xCB})\\ \Delta R_{4}=\cos(C)\cdot (q_{2}\cdot \delta_{\gamma BM}-h_{1}\cdot q_{2}\cdot \alpha_{BM}-h_{1}\cdot q_{2}\cdot \alpha_{CB})+\sin(C)\cdot (-q_{2}\cdot \delta_{xCB}-q_{2}\cdot \delta_{xBM}-h_{1}\cdot q_{2}\cdot \beta_{BM}) \end{array}\right.$$

After grouping constant, cosine and sine terms from the four ΔR expressions in Equation (18), all ball-bar length formulas are rearranged into the unified form listed in Equation (19) by substituting the fundamental relation L=R+ΔR, which yields the explicit length expressions for the four measurement modes as follows:

(19) $$\left\{\begin{array}{l} L_{1}=R+\Delta R_{1}=R+\cos(B)\cdot (-q_{0}\cdot \delta_{xBM})+\sin(B)\cdot (q_{0}\cdot \delta_{zBM})-q_{0}\cdot \delta_{xCB}\\ \begin{array}{l} L_{2}=R+\Delta R_{2}=R+\cos(B)\cdot (-q_{1}\cdot \delta_{xBM}+q_{1}\cdot L_{0}\cdot \gamma_{BM})\\ \;\;\;\;\;+\sin(B)\cdot (q_{1}\cdot \delta_{zBM}+q_{1}\cdot L_{0}\cdot \alpha_{BM})-q_{1}\cdot \delta_{xCB} \end{array}\\ L_{3}=R+\Delta R_{3}=R+\cos(C)\cdot (q_{2}\cdot \delta_{yBM})+\sin(C)\cdot (-q_{2}\cdot \delta_{xBM}-q_{2}\cdot \delta_{xCB})\\ \begin{array}{l} L_{4}=R+\Delta R_{4}=R+\cos(C)\cdot (q_{2}\cdot \delta_{yBM}-h_{1}\cdot q_{2}\cdot \alpha_{BM}-h_{1}\cdot q_{2}\cdot \alpha_{CB})\\ \;\;\;\;\;+\sin(C)\cdot (-q_{2}\cdot \delta_{xCB}-q_{2}\cdot \delta_{xBM}-h_{1}\cdot q_{2}\cdot \beta_{BM}) \end{array} \end{array}\right.$$

Based on Equation (19), the relationship between the eight PIGEs of the rotating axes and *L* under the four measurement modes can be uniformly summarized as follows:

(20) $$L=R+\Delta R=R+e_{0}{+e}_{1}\cdot \cos(\theta )+e_{2}\cdot \sin(\theta )$$

#### 3.3.3. Numerical Verification of the Linearization Error

To verify that the neglected higher-order terms in Equations (13)–(16) have no significant impact, a numerical comparison is performed using the simulation parameters in this section (R=100 mm, $h_{0}=25.5$ mm, $q_{0}=0.967$). The PIGEs adopted in this simulation are set according to the actual accuracy level of industrial five-axis machine tools and in accordance with the tolerance requirements specified in ISO 10791-1 [5]. The positional errors of the rotary axes are set to 100 μm ($\delta_{xBM}=\delta_{yBM}=\delta_{zBM}=\delta_{xCB}$), and the angular errors are set to 0.005° ($\gamma_{BM}=\alpha_{BM}=\alpha_{CB}=\beta_{BM}$). Both values are within the typical geometric error ranges of industrial-grade machine tools.

Two BB length datasets are generated for all four modes:

(1) Full model: Equations (6)–(12) with all second-order error terms retained in the matrix chain multiplication and the distance formula (exact trigonometric functions for all error angles, no small-angle approximations).

(2) Linearized model: Equation (18) with only first-order terms retained.

The maximum absolute deviation between the full and linearized models is less than 0.01 μm, and the relative deviation with respect to the peak error-induced BB length variation is below 0.01%. These deviations are far below the measurement resolution of the Renishaw QC20-W ball bar (0.1 μm) and the identification accuracy requirement for high-precision machining (1 μm). Therefore, the first-order approximation in Equations (13)–(16) is numerically safe and will not introduce observable influence on the PIGE identification results.

After verifying the accuracy of the linearization approximation, further parametric simulation is performed to analyze the physical meaning of $e_{0}$, $e_{1}$, and $e_{2}$. In Equation (20), $e_{0}$, $e_{1}$, and $e_{2}$ are variables representing the PIGEs of the rotating axes. Specifically, $e_{0}$ denotes the fitted circle’s radius deviation, which corresponds to the average variation in ball-bar length induced by geometric errors, while $e_{1}$ and $e_{2}$ represent the center offsets in two orthogonal directions within the measurement plane. This unified expression lays a theoretical foundation for decoupling PIGEs using least-squares circle fitting on measured ball-bar data.

**Figure 5:**
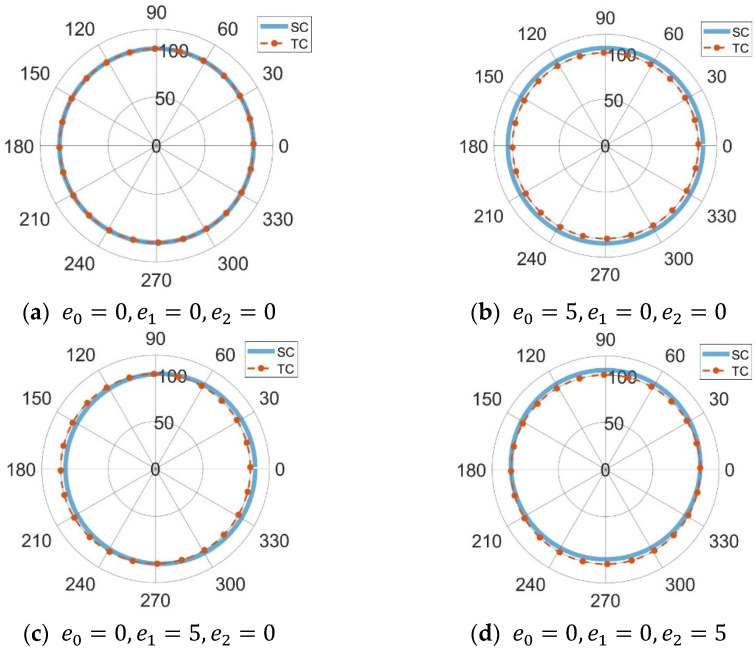
Influence of variables $e_{0}$, $e_{1}$, and $e_{2}$ on *L*.

By simulating and analyzing the effects of $e_{0}$, $e_{1}$, and $e_{2}$ on *L*, this paper explores how different PIGEs influence *L* across the four modes. MATLAB R2018b simulations were used to set R to 100 mm, assign values of 0 or 5 mm to variables $e_{0}$, $e_{1}$, and $e_{2}$, and investigate how these different values influence the measurement length L of the BB. The simulation results are shown in Figure 5.

As can be seen from Figure 5, when $e_{0}$, $e_{1}$, and $e_{2}$ are all zero, the simulated circle (SC) of the BB coincides with the theoretical circle (TC). When $e_{0}=5$ mm and $e_{1}=e_{2}=0$, the radius of SC deviates from that of TC while the circle centers remain coincident. When $e_{0}=5$ and either $e_{1}$ or $e_{2}$ is 5 mm, SC and TC have identical radii but their centers exhibit horizontal or vertical offsets, respectively.

Analytical derivation: Equation (20) can be rewritten as $\Delta R=L-R=e_{0}+e_{1}\cos\theta +e_{2}\sin\theta$. Here, $e_{0}$ is a constant term independent of θ, which only causes a uniform variation in the trajectory radius; $e_{1}\cos\theta +e_{2}\sin\theta$ is a harmonic term, and its composite amplitude $\sqrt{e_{1}^{2}+e_{2}^{2}}$ determines the eccentricity of the circle center in the radial plane. Specifically, the radius deviation is solely determined by $e_{0}$ ($\Delta r=e_{0}$), and the center offset is determined by the vector ($e_{1}$, $e_{2}$), whose magnitude is defined as $\Delta c=\sqrt{e_{1}^{2}+e_{2}^{2}}$. Since these two effects are mathematically independent, $e_{0}$ and ($e_{1}$, $e_{2}$) are completely decoupled, independently controlling the radius deviation and center offset, respectively.

Numerical sensitivity analysis: To further quantitatively verify the above analytical conclusions, single-variable control experiments were conducted under the conditions of R=100 mm and $\theta \in \left[0^{{}^{\circ}},360^{{}^{\circ}}\right]$. Table 2 lists the quantitative results of the circle trajectory geometric characteristics under different parameter combinations. As shown in the table, when only $e_{0}$ is non-zero, the radius change equals $e_{0}$ while the center offset is zero; when only $e_{1}$ or $e_{2}$ is non-zero, the center shifts along the X or Y direction by the corresponding amount while the radius remains unchanged; when multiple parameters coexist, the radius change and center offset still maintain independent superposition. In summary, through the radius deviation and center eccentricity obtained from measurement data fitting, the precise decoupling and identification of $e_{0}$ and ($e_{1}$, $e_{2}$) can be achieved.

#### 3.3.4. Principle of PIGE Decoupling Based on Circle Fitting

During measurement, the tool ball $O_{T}$ remains stationary, with its installation errors being ignored. Installation errors of the workpiece ball $O_{W}$ significantly affect the circle’s radius fitted from the BB data. To minimize the impact of these installation errors, only the center deviation derived from the BB data is utilized to decouple various errors.

When measuring in mode 1, it can be inferred from $L_{1}$ in Equation (19) that: $-q_{0}\cdot \delta_{xBM}=e_{x1}$ and $q_{0}\cdot \delta_{zBM}=-e_{z1}$. Therefore,

(21) $$\delta_{xBM}=-\frac{e_{x1}}{q_{0}}$$

(22) $$\delta_{zBM}=-\frac{e_{z1}}{q_{0}}$$

When measuring in mode 2, it can be inferred from $L_{2}$ in Equation (19) that $-q_{1}\cdot \delta_{xBM}+q_{1}\cdot L_{0}\cdot \gamma_{BM}=e_{x2}$ and $q_{1}\cdot \delta_{zBM}+q_{1}\cdot L_{0}\cdot \alpha_{BM}=-e_{z2}$. Therefore,

(23) $$\gamma_{BM}=\frac{e_{x2}+q_{1}.\delta_{xBM}}{q_{1}.L_{0}}$$

(24) $$\alpha_{BM}=\frac{e_{z2}+q_{1}.\delta_{zBM}}{-q_{1}.L_{0}}$$

When measuring in mode 3, it can be inferred from $L_{3}$ in Equation (19) that $q_{2}\cdot \delta_{xBM}+q_{2}\cdot \delta_{xCB}=e_{x3}$ and $q_{2}\cdot \delta_{yBM}=e_{y3}$. Therefore,

(25) $$\delta_{yBM}=\frac{e_{y3}}{q_{2}}$$

(26) $$\delta_{xCB}=\frac{e_{x3}-q_{2}.\delta_{xBM}}{q_{2}}$$

When measuring in mode 4, it can be inferred from $L_{4}$ in Equation (19) that $q_{2}.\delta_{yBM}-h_{1}.q_{2}.\alpha_{BM}-h_{1}.q_{2}.\alpha_{CB}=e_{y4}$ and $-q_{2}.\delta_{xCB}-q_{2}.\delta_{xBM}-h_{1}.q_{2}.\beta_{BM}=-e_{x4}$.

Therefore,

(27) $$\alpha_{CB}=\frac{e_{y4}-q_{2}.\delta_{yBM}+h_{1}.q_{2}.\alpha_{BM}}{-h_{1}.q_{2}}$$

(28) $$\beta_{BM}=\frac{e_{x4}-q_{2}.\delta_{xCB}-q_{2}.\delta_{xBM}}{h_{1}.q_{2}}$$

where $e_{x1},e_{z1},e_{x2},e_{z2},e_{x3},e_{y3},e_{x4}$ and $e_{y4}$ respectively represent the circle’s center deviations fitted from the BB data in the X-Z, X-Z, X-Y, and X-Y axis directions for four measurement modes.

The decoupling of the eight PIGEs relies on the independent linear equations established by the four measurement modes using Equations (21)–(28). Each mode provides two independent equations derived from the circle center offsets, yielding eight equations in total. The coefficient matrix of the identification model is constructed to be full rank (rank = 8), which eliminates rank deficiency and multicollinearity. Since the number of independent equations matches the number of unknown errors, the identification result is theoretically unique. No redundant or conflicting constraints exist in the solution process, ensuring stable and reliable error identification.

As mentioned above, the eight PIGEs can be decoupled by Equations (21)–(28). In the measurement process, only the single rotation axis rotates, which avoids errors introduced by the movement of other axes, thereby achieving accurate measurement results and simplifying the measurement process.

## 4. Experiment on Identification and Compensation of the PIGEs

### 4.1. Measurement Experiment of the PIGEs

#### 4.1.1. Experimental Setup and Test Conditions

In this experiment, the Haas UMC500 five-axis machine tool was used to measure the PIGEs of the rotating axes. The structure of the machine tool is shown in Figure 1. The Renishaw QC20-W ball bar (BB, calibrated prior to measurement) was selected as the measuring instrument for error testing. Since the spindle remains stationary under the measurement modes proposed in this study, accurate identification of PIGEs requires precise determination of the rotation centers of the two rotary axes, as detailed in Ref. [28].

To ensure that measured deviations mainly reflect position-independent geometric errors (PIGEs) rather than dynamic or servo-induced errors, all four measurement modes were implemented with identical CNC parameter settings. The B- and C-axes operated under single-axis rotary feed at a feed rate of 1°/s, while the linear X, Y and Z axes were fully locked. This feed speed is far lower than the maximum rapid traverse speed of the Haas UMC-500 (50°/s for both B- and C-axes), thereby minimizing inertial effects, servo mismatch and thermal drift throughout data acquisition. The machine is equipped with a Haas CNC system, and all servo parameters were kept at factory default values to guarantee consistent and repeatable measurement conditions. During full rotational sweep measurements, the Renishaw QC20-W ball bar collected data at a sampling frequency of 1000 Hz.

#### 4.1.2. Measurement Procedures for Four Modes and Identified PIGE Results

**Figure 6:**
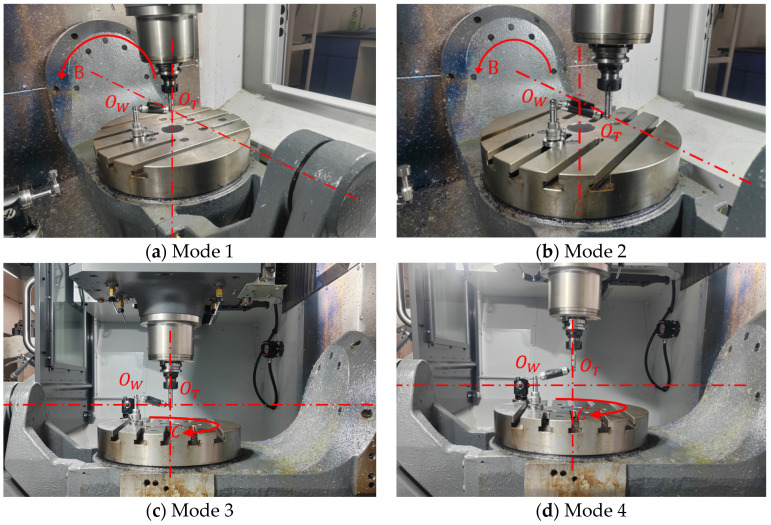
Actual measurement diagram.

The experiment was performed following the four PIGE measurement modes described above, with ambient temperature kept constant at 20 °C. The on-site fixture setup is presented in Figure 6.

Mode 1

(1) Set the rotation axes of B and C to their respective zero positions, i.e., B=0,C=0;

(2) Mount the workpiece ball $O_{W}$ at the position (−96.7 mm, 0, 25.5 mm) in MCS.

(3) Set the B-axis to rotate from −15° to 60°, with the C-axis remaining stationary. Due to the BB being installed at an angle on the C rotary table’s surface, the actual workpiece ball $O_{W}$ rotates from −29.76° to 45.24° around the B-axis. After the measurement, adjust the B rotary table to the horizontal position, i.e., B=0.

Mode 2

(1) Place the rotation axes at their respective zero positions, and move $O_{T}$ to the position (0, −96.7 mm, 0) in MCS.

(2) The C rotary table drives the workpiece ball $O_{W}$ to rotate from 0° to −30°.

(3) Set the B-axis to rotate from −15° to 60°, and $O_{W}$ rotates around the B-axis. After the measurement, move $O_{T}$ back to (0,0,0) in MCS, and adjust the B rotary table to the horizontal position, i.e., B=0.

Mode 3

(1) Place the rotation axes of B and C at their respective zero positions, and the workpiece ball $O_{W}$ is placed at the position (0, −96.7 mm, 25.5 mm) in MCS.

(2) Keep the B-axis stationary while the C rotary table drives $O_{W}$ to rotate from 0° to 360°. After the measurement, adjust the C rotary table back to its original position.

Mode 4

(1) Place the rotation axes of B and C at their respective zero positions, and $O_{W}$ is placed at the position (0, −96.7 mm, 25.5 mm) in MCS; $O_{T}$ is moved forward along the Z-axis to the position (0, 0, 51 mm) in MCS.

(2) Follow the same measurement procedure as in step (2) of Mode 3.

**Figure 7:**
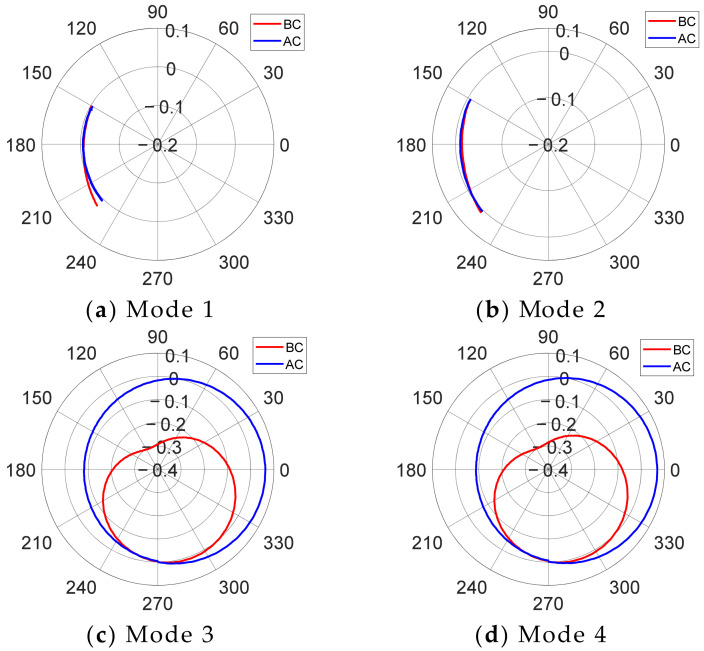
Measurement data from the BB before and after compensation.

The above procedures are performed repeatedly for five times and the BB data is recorded. According to the data, the least squares method is used to fit the motion trajectory of $O_{W}$ under the four modes, and the eight PIGEs are decoupled. The motion trajectory of $O_{W}$ before compensation under the four modes is shown in Figure 7. The eight PIGEs for the rotating axes are presented in Table 3.

### 4.2. Compensation of the PIGEs

In this study, all four measurement modes adopt locked linear axes with no coordinated motion. Consequently, the geometric errors of linear axes are negligible to the tool tip pose, and only eight PIGEs of the rotary axes are considered during modeling. The volumetric ideal kinematic model of the machine tool is first established, followed by the construction of the volumetric geometric error model by incorporating rotary-axis error terms. Pose compensation values are then derived, and error compensation is ultimately implemented via offline NC code modification.

**Figure 8:**
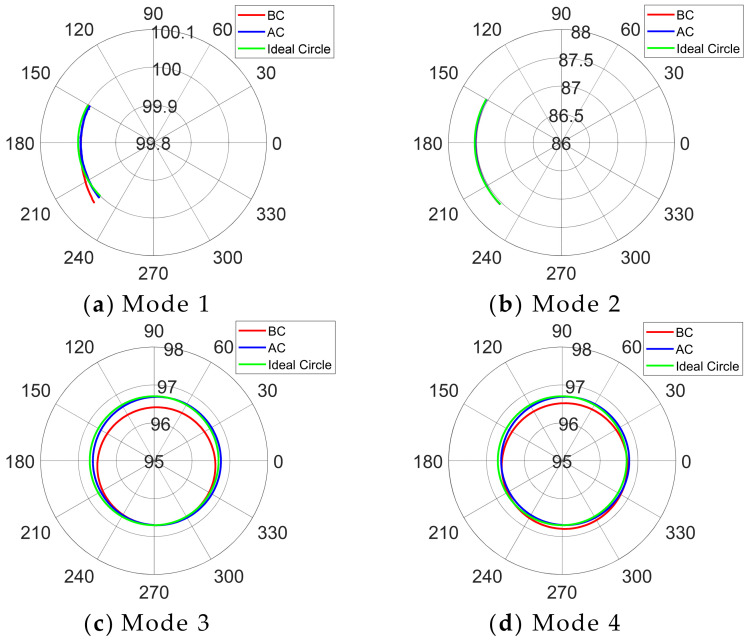
Trajectory of workpiece ball before and after compensation with ideal trajectory.

The four measurement modes are re-executed using the modified NC codes, and the experimental data are collected and recorded accordingly. The data from the BB and the motion trajectories of $O_{W}$ under the four measurement modes with compensation are fitted, as shown in Figure 7 and Figure 8. In the figures, BC stands for “Before compensation,” and AC stands for “After compensation.”

In mode 1 and mode 2, the measured value after compensation is closer to the ideal value (zero line) than the measured value before compensation. In mode 3 and mode 4, the measured value after compensation is less than that of the BB before compensation. Therefore, the proposed method can accurately identify geometric errors with high reliability. Table 4 presents the compensated PIGEs.

The deviation between the motion trajectory of the workpiece ball after compensation and the ideal value in the four modes is significantly smaller than the deviation before compensation, as shown in Figure 8.

### 4.3. Result Analysis

After compensation, the maximum individual error $\delta_{yBM}$, defined as the Y-axis positional deviation in the B-axis with respect to the machine bed, is reduced from 144.53 μm to 7.72 μm with a prominent improvement ratio of 94.6%. The remarkable accuracy enhancement further validates the reliability of the error identification results. The overall error (positional error) improvement rate in this study reaches 79.48%.

To verify the repeatability and reliability of the experimental results, five repeated measurements were performed for both uncompensated and compensated conditions. The measured values before compensation were 140.90, 142.88, 144.53, 146.12, and 148.10 μm, with a sample standard deviation of 2.79 μm; the measured values after compensation were 7.25, 7.55, 7.72, 7.89, and 8.19 μm, with a sample standard deviation of 0.35 μm. According to the instrument uncertainty of Renishaw QC20-W ball bar (±(0.7 + 0.3%L) μm, k = 2, L = 100 mm), the expanded measurement uncertainties are ±5.92 μm (k = 2) before compensation and ±2.12 μm (k = 2) after compensation. The small standard deviation and expanded uncertainty confirm the high stability and credibility of the experimental results.

To quantitatively evaluate the performance of the proposed method, five standardized evaluation indicators widely adopted in the field of machine tool geometric error detection are summarized in Table 5, including maximum original error, maximum residual error after compensation, overall error improvement rate, as well as the repeatability and expanded measurement uncertainty evaluated at the worst-measurement point. These quantitative results fully reflect the compensation capability, measurement stability and credibility of the developed PIGE identification scheme.

In terms of technical implementation, this method adopts a standard fixed-length ball bar without additional auxiliary fixtures, which effectively eliminates installation-induced errors. During each measurement, all linear axes are fully locked and only a single rotary axis is driven to move independently. This design realizes complete decoupling of geometric errors caused by multi-axis linkage, simplifying the measurement process and improving on-site practicability.

In summary, the proposed single-axis rotation measurement scheme can accurately identify and compensate the eight position-independent geometric errors of rotary axes on BC-type dual rotary table five-axis machine tools. The quantitative results from multiple standard indicators verify the effectiveness and practicability of this method.

## 5. Conclusions

(1) A single-axis-rotation-based double ball-bar scheme is proposed to identify PIGEs of rotary axes. On the basis of homogeneous coordinate transformation and the measurement principle of BB, the developed approach fixes all translational axes and enables single rotary-axis motion in each test, thereby eliminating linear-axis-induced geometric error coupling. Mathematical correlations between PIGEs and BB length deviations are further formulated for the four developed measurement configurations.

(2) The impact of GEs on the BB length was analyzed through simulation, and the eccentricity of the fitted circle from the BB data was proposed as an approach to identify the eight PIGEs. Through the error identification experiment, the eight PIGEs were identified.

(3) A set of standardized evaluation indicators is used to quantitatively assess the performance of the proposed method. Experimental results show that the maximum residual value of individual position-independent geometric errors decreases to 7.72 μm after compensation, and the overall error improvement rate reaches 79.48%. The small standard deviation and expanded measurement uncertainty obtained from repeated measurements verify the high stability and reliability of the identification and compensation process. Benefiting from the design of fully locked linear axes and independent single-axis rotation, the proposed method realizes complete decoupling of geometric errors. Meanwhile, the adoption of standard fixed-length ball bar without auxiliary fixtures effectively reduces installation errors, which guarantees good practicability for the PIGE identification of BC-type dual rotary table five-axis machine tools.

## Figures and Tables

**Table 1:** Homogeneous coordinate transformation matrix.

ET/RA	HCTM	ET/RA	HCTM
$\delta_{xBM}$	$D^{1}\left(\delta_{xBM}\right)=\left[\begin{array}{cccc} 1 & 0 & 0 & \delta_{xBM}\\ 0 & 1 & 0 & 0\\ 0 & 0 & 1 & 0\\ 0 & 0 & 0 & 1 \end{array}\right]$	$\beta_{BM}$	$D^{5}\left(\beta_{BM}\right)=\left[\begin{array}{cccc} C_{\beta_{BM}} & 0 & S_{\beta_{BM}} & 0\\ 0 & 1 & 0 & 0\\ -S_{\beta_{BM}} & 0 & C_{\beta_{BM}} & 0\\ 0 & 0 & 0 & 1 \end{array}\right]$
$\delta_{zBM}$	$D^{3}\left(\delta_{zBM}\right)=\left[\begin{array}{cccc} 1 & 0 & 0 & 0\\ 0 & 1 & 0 & 0\\ 0 & 0 & 1 & \delta_{zBM}\\ 0 & 0 & 0 & 1 \end{array}\right]$	$\alpha_{BM}$	$D^{4}\left(\alpha_{BM}\right)=\left[\begin{array}{cccc} 1 & 0 & 0 & 0\\ 0 & C_{\alpha_{BM}} & -S_{\alpha_{BM}} & 0\\ 0 & S_{\alpha_{BM}} & C_{\alpha_{BM}} & 0\\ 0 & 0 & 0 & 1 \end{array}\right]$
$\delta_{yBM}$	$D^{2}\left(\delta_{yBM}\right)=\left[\begin{array}{cccc} 1 & 0 & 0 & 0\\ 0 & 1 & 0 & \delta_{yBM}\\ 0 & 0 & 1 & 0\\ 0 & 0 & 0 & 1 \end{array}\right]$	$\gamma_{BM}$	$D^{6}\left(\gamma_{BM}\right)=\left[\begin{array}{cccc} C_{\gamma_{BM}} & -S_{\gamma_{BM}} & 0 & 0\\ S_{\gamma_{BM}} & C_{\gamma_{BM}} & 0 & 0\\ 0 & 0 & 1 & 0\\ 0 & 0 & 0 & 1 \end{array}\right]$
$\delta_{xCB}$	$D^{1}\left(\delta_{xCB}\right)=\left[\begin{array}{cccc} 1 & 0 & 0 & \delta_{xCB}\\ 0 & 1 & 0 & 0\\ 0 & 0 & 1 & 0\\ 0 & 0 & 0 & 1 \end{array}\right]$	$\alpha_{CB}$	$D^{4}\left(\alpha_{CB}\right)=\left[\begin{array}{cccc} 1 & 0 & 0 & 0\\ 0 & C_{\alpha_{CB}} & -S_{\alpha_{CB}} & 0\\ 0 & S_{\alpha_{CB}} & C_{\alpha_{CB}} & 0\\ 0 & 0 & 0 & 1 \end{array}\right]$
B	$D^{5}\left(B\right)=\left[\begin{array}{cccc} C_{B} & 0 & S_{B} & 0\\ 0 & 1 & 0 & 0\\ -S_{B} & 0 & C_{B} & 0\\ 0 & 0 & 0 & 1 \end{array}\right]$	C	$D^{6}\left(C\right)=\left[\begin{array}{cccc} C_{C} & -S_{C} & 0 & 0\\ S_{C} & C_{C} & 0 & 0\\ 0 & 0 & 1 & 0\\ 0 & 0 & 0 & 1 \end{array}\right]$

**Table 2:** Quantitative relationship between simulation parameters and circle trajectory geometric characteristics.

Parameter Combination	$e_{0}$ (mm)	$e_{1}$ (mm)	$e_{2}$ (mm)	Radius Change (mm)	Center Offset (mm)
Ideal (no error)	0.0	0.0	0.0	0.0	0.0
Only $e_{0}$ non-zero	5.0	0.0	0.0	5.0	0.0
Only $e_{1}$ non-zero	0.0	5.0	0.0	0.0	5.0
Only $e_{2}$ non-zero	0.0	0.0	5.0	0.0	5.0
$e_{0}+e_{1}$ combined	5.0	5.0	0.0	5.0	5.0
$e_{0}+e_{2}$ combined	5.0	0.0	5.0	5.0	5.0
$e_{1}+e_{2}$ combined	0.0	3.0	4.0	0.0	5.0
All combined	5.0	3.0	4.0	5.0	5.0

**Table 4:** Values of PIGEs after compensation.

Error Term	$\delta_{xBM}$ (μm)	$\delta_{zBM}$ (μm)	$\gamma_{BM}$ (°)	$\alpha_{BM}$ (°)	$\delta_{yBM}$ (μm)	$\delta_{xCB}$ (μm)	$\alpha_{CB}$ (°)	$\beta_{BM}$ (°)
Error value	−34.46	2.57	0.0056	0.0038	7.72	2.34	0.0030	0.0045

**Table 5:** Standardized quantitative evaluation results of the proposed method.

Evaluation Index	Value
Maximum original PIGE (μm)	144.53
Maximum residual PIGE after compensation (μm)	7.72
Overall error improvement rate	79.48%
Sample standard deviation before compensation (μm)	2.79
Sample standard deviation after compensation (μm)	0.35
Expanded measurement uncertainty before compensation (μm)	±5.92
Expanded measurement uncertainty after compensation (μm)	±2.12

Note: All data are obtained from repeated experiments on the BC-type dual rotary table five-axis machine tool in this study.

**Table 3:** Identified results of the 8 PIGEs.

Error Term	$\delta_{xBM}$ (μm)	$\delta_{zBM}$ (μm)	$\gamma_{BM}$ (°)	$\alpha_{BM}$ (°)	$\delta_{yBM}$ (μm)	$\delta_{xCB}$ (μm)	$\alpha_{CB}$ (°)	$\beta_{BM}$ (°)
Error value	−86.54	9.06	0.0081	0.0039	144.53	28.26	0.0012	0.0062

## Data Availability

The data presented in this study are available on reasonable request from the corresponding author.
